# Influenza B-Associated Mild Encephalopathy with Reversible Splenial Lesion in an Adult: A Case Report

**DOI:** 10.3390/neurolint17120194

**Published:** 2025-11-30

**Authors:** Nicodemus Edrick Oey, Moe Pearl Shwe, Alvin Dingyuan Wang, Andrew Che Fai Hui

**Affiliations:** 1 Division of Rehabilitation Medicine, Department of Medicine, National University Hospital, 1E Kent Ridge Road, NUHS Tower Block, Singapore 119228, Singapore; nicodemus_edrick_oey@nuhs.edu.sg; 2 Division of Neurology, Department of Medicine, Ng Teng Fong General Hospital, 1 Jurong East Street 21, Singapore 609606, Singapore; drmoepearlshwe@gmail.com; 3 Department of Microbiology, Centre for Laboratory Medicine, Leeds Teaching Hospitals NHS Trust, St James University Hospital, Beckett Street, Leeds LS9 7TF, UK; dingyuan.wang@nhs.net

**Keywords:** MERS, splenium, influenza B, corpus callosum, infection

## Abstract

Background/Objectives: Mild Encephalopathy with Reversible Splenial Lesion (MERS) is a potential complication of certain viral infections, but adult cases involving influenza are rare in the literature. Here, we report a case of a 31-year-old Chinese gentleman with an atypical presentation of Influenza B-associated mild encephalopathy with reversible splenial lesion (MERS). Methods: This is a case report with a detailed chronology followed by a discussion of pathophysiology. Results: The patient presented acutely to the tertiary hospital with a severe headache and a peculiar automatism pattern of behaviour involving intermittent screaming, involuntary jerking movements of the upper limbs, and incoherent speech, which culminated in an episode of tonic–clonic seizure lasting 3 min. Symptoms started on the day that the patient was diagnosed with Influenza B and given the antiviral Baloxavir by his GP. Clinically, there was high anion gap metabolic acidosis with hyperlactatemia, rhabdomyolysis, hepatitis transaminitis and absolute lymphopenia. Nasopharyngeal swab PCR and immunofluorescence was positive for Influenza B. EEG was normal, but an MRI of the brain showed a cytotoxic lesion of the splenium of the corpus callosum. The patient was started on Oseltamivir and made a complete neurological recovery, with a repeat MRI showing resolution of the splenial lesion at 3 months. MERS is a rare clinic-radiological syndrome characterized by a transient encephalopathy and a reversible lesion in the splenium of the corpus callosum, which has been reported mostly in the pediatric population. Conclusions: This case report of an influenza B-triggered MERS in an adult highlights the importance of maintaining MERS as a differential for acute encephalopathy in adults with a viral prodrome.

## 1. Case Presentation

A 31-year-old Chinese gentleman, a teetotaler with no past medical history, presented with one day of acute respiratory tract symptoms. Aside from a travel history to London a month prior and to Batam, Indonesia, a week prior, there were no reported sick contacts, and he denied illicit drug usage. He started feeling unwell with generalized lethargy and flu symptoms on Day 1, but did not have any fever. On Day 2, he developed a fever with chills, together with a headache. A GP attendance yielded a serological diagnosis of Influenza B, and he was prescribed the antiviral agent Baloxavir. In the evening of that day, he became delirious and unable to respond meaningfully to questions. En route to the hospital, the patient had a generalized tonic–clonic seizure, which lasted for two minutes and self-aborted.

**Figure 1:**
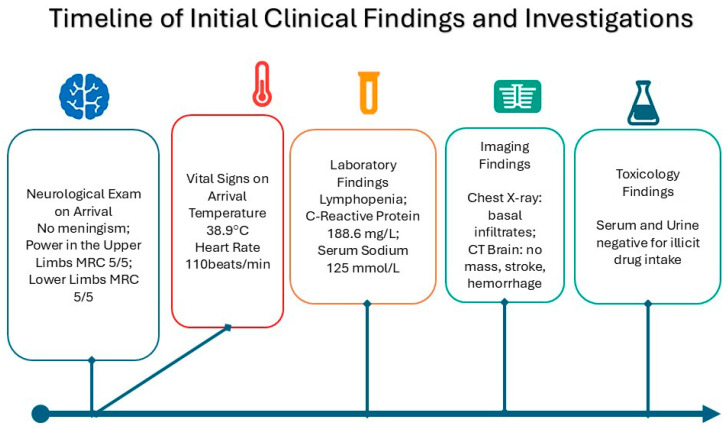
Clinical presentation and sequence of events.

Upon arrival in the general ward, the patient was febrile with a temperature of 38.9 C, heart rate was tachycardic at 110 beats/min. Clinically, he was confused and not oriented to time, place, or person. A full neurological examination showed that the patient had no signs of meningism; muscle power was MRC grade 5/5 in the upper limbs and 4+/5 in the lower limbs due to pain over the proximal muscles. Tone, reflexes, gait, and sensory examination were unremarkable (Figure 1).

Initial blood tests were unremarkable apart from lymphopenia on the blood counts, an elevated C-Reactive Protein level of 168.6 mg/L, and hyponatremia of Na 125 mmol/L. Arterial Blood Gas showed a mixed metabolic acidosis, which was partially compensated by acute respiratory alkalosis. Chest X-ray on arrival showed infiltrates in the basal regions of the lungs. CT scan of the brain was negative for masses, stroke, or hemorrhages. Serum and urine toxicology were negative for any illicit drug intake.

## 2. Diagnosis

The constellation of fever, altered mental state, and seizures strongly suggests possible meningoencephalitis, which has a broad differential list including metabolic, drug-related, infective (bacterial, viral, fungal, parasitic), autoimmune, and paraneoplastic causes. The tempo of the patient’s initial deterioration pointed toward an infective cause as being more likely (Table 1). The lack of meningism signs indicates a cerebral pathology without meningeal involvement; hence, meningitis was not likely, while the viral prodrome with no elevated white cell count narrowed the list down to viral encephalopathy. As such, the patient was investigated for multiple infective etiologies, including HIV, Hepatitis B and C, Syphilis, Malaria, Dengue flavivirus, Leptospirosis, Mycoplasma, Rickettsia, Zika virus, which were all negative. The confirmed detection of Influenza B virus in the nasopharyngeal swab Polymerase Chain Reaction (PCR) using primers directed against the Nucleoprotein of Influenza B, combined with the transient MRI finding of a Diffusion Weighted Imaging (DWI) signal abnormality in the splenium of the corpus callosum, solidified the diagnosis of MERS associated with acute Influenza B.

## 3. Initial Management and Prognosis

**Figure 2:**
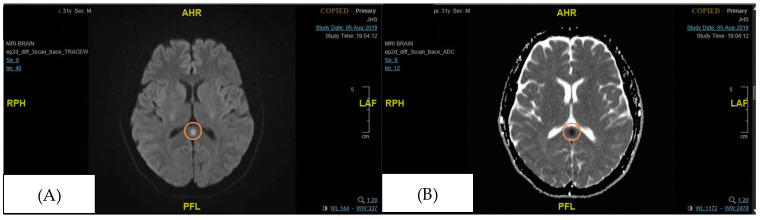
Focal signal abnormality in the splenium of the corpus callosum (initial MRI brain performed during admission). The orange circle signifies the area of the splenium that had high signal intensity. (**A**) DWI (Diffusion Weighted Imaging), (**B**) ADC (Apparent Diffusion Coefficient).

At the Emergency Department, the patient was initiated on broad-spectrum antibiotics and antiviral therapy at meningitic doses in anticipation of feared meningoencephalitis, as well as Intravenous (IV) Levetiracetam to control seizures. From the outset, we strongly recommended Lumbar Puncture for Cerebrospinal Fluid (CSF) analysis to look for any additional cause for his clinical seizures; however, the patient’s family persistently declined any invasive investigation. The patient eventually consented to an Electroencephalogram, which was a normal recording, and a contrasted MRI of the brain (Figure 2), which eventually clinched the diagnosis of MERS associated with Influenza B. The patient’s clinical features were consistent with symptoms of MERS that have been reported previously (Table 2). An early Infectious Disease consult was made, which provided guidance for the off-label use of Oseltamivir at 150 mg twice a day for two weeks. Given the rarity of available case reports, the neurological manifestations of MERS are known to be widely variable, but full recovery was expected in most cases within days to weeks.

## 4. Case Progression and Outcome

On Day 3, the patient’s mental status improved to being oriented to time, place, and person, and being able to answer relevantly despite an ongoing low-grade temperature of 37.8 °C. Transient delirium was confirmed as he was unable to recall the event and the reason for admission. Physically, he reported a new symptom of pain in both thighs, which was attributed to rhabdomyolysis, as his Creatine Kinase (CK) levels peaked at more than 42,670 u/L, well beyond the limits of detection in our laboratory test. Aggressive intravenous hydration was commenced, and by day 4, the patient’s behaviour and mental state had completely recovered, with no seizure documented in the general ward. Vital signs remained stable with a temperature of 37.6 °C, heart rate 70-88 beats/min, SpO_2_ 98%, with no focal neurological deficits on examination. IV Ampicillin was stopped, IV Ceftriaxone was deescalated to Co-amoxiclav for presumed pneumonia, aimed for a total duration of 7 days, and he completed 7 days of IV acyclovir as well. IV levetiracetam was changed to oral form 500 mg BD, continued for 1 month, and then stopped.

**Figure 3:**
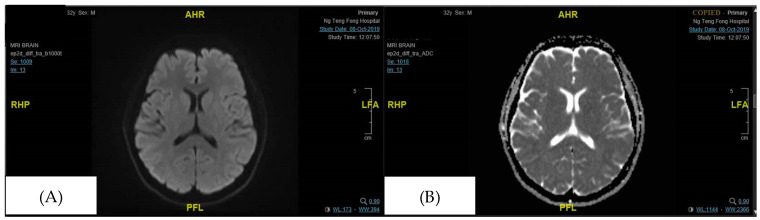
The focus of DWI signal abnormality in the splenium of the corpus callosum is no longer seen (in the follow-up visit 3 months later). (**A**) DWI (Diffusion Weighted Imaging), (**B**) ADC (Apparent Diffusion Coefficient).

The patient was successfully discharged on Day 8 of illness, with CK levels falling to 294 u/L on the day of discharge. MRI Brain that was repeated in clinic 3 months later showed a complete resolution of the previously noted focus of DWI signal abnormality in the splenium of the corpus callosum (Figure 3), and the patient remained well without neurological sequelae.

## 5. Discussion and Conclusions

MERS (Mild Encephalopathy with a Reversible Splenial Lesion) is a clinico-radiological entity that presents with acute encephalopathy and a characteristic Diffusion Weighted Imaging (DWI) signal abnormality in the splenium of the corpus callosum [1]. Patients may report prodromal symptoms such as fever, cough, diarrhea, vomiting, and headache, followed by neurological symptoms including reduced consciousness level, behavioural changes, drowsiness, seizures, headache, speech disturbances, visual hallucinations, transient blindness, monoparesis, or ataxia, especially in pediatric cases [2]. In this case, we report an adult patient with Influenza B infection who presented with delirium and seizures, which were subsequently attributed to MERS.

Though one other study has reported a case of MERS in a 50-year-old man with prodromal flu [3], we note no other adult cases implicating Influenza B as a potential causative agent of MERS [3,4]. Other causative agents that have been reported include respiratory syncytial virus and Pseudomonas putida [5], rotavirus [6], as well as Mycoplasma pneumoniae [7].

The severe systemic inflammation observed in the patient, characterized by high anion gap metabolic acidosis, hyperlactatemia, rhabdomyolysis, hepatitis transaminitis, and absolute lymphopenia, is the hallmark of a cytokine storm. A case report of an adult with Influenza A-associated MERS demonstrated markedly elevated levels of pro-inflammatory cytokines, specifically interleukin-6 (IL-6) and interleukin-10 (IL-10), in both serum and cerebrospinal fluid (CSF) [4]. These cytokine levels normalized as the patient recovered, providing direct biochemical evidence that a systemic inflammatory response, rather than direct viral invasion of the brain, is the primary driver of the splenial lesion. The cytokine storm can lead to a breakdown of the blood–brain barrier, resulting in vasogenic edema within the tightly packed myelin fibres of the splenium, which explains the transient diffusion restriction observed on MRI (Figure 2 and Figure 3).

The corpus callosum is the largest white matter bundle in the brain, with projections into prefrontal, premotor, primary motor, and primary sensory areas. Disturbances in callosal connections can cause disorders of motor control, spatial orientation, vision, hearing, and language-related behaviour [4]. These may explain the neurological symptoms of MERS. The characteristic MRI findings of MERS are a reversible diffusion restriction in the splenium of the corpus callosum, with a corresponding hypodensity in the apparent diffusion coefficient (ADC) image [5]. There are two radiological types of MERS based on MRI: type 1, where the lesion is limited to the splenium of the corpus callosum, and type 2, where the lesions extend to the entire corpus callosum or symmetrically extend to the adjacent white matter. MERS type 1, as reported in this case, is more common and carries a more favourable outcome than type 2, though the rarity of the diagnosis precludes epidemiological correlation [6].

The patient’s hyponatremia (serum sodium 125 mmol/L) is a critical component of the clinical picture. A study published in *Brain & Development* established a strong association between hyponatremia and MERS, finding that patients with MERS had a significantly lower mean serum sodium level (131.0 ± 4.1 mmol/L) compared to healthy controls [8]. The authors postulate that acute hypotonic hyponatremia causes water to enter the brain, leading to intramyelinic edema in the corpus callosum, which is rich in myelin. This mechanism is consistent with the cytotoxic edema pattern seen on MRI. The presence of hyponatremia in this patient provides a plausible and well-supported explanation for the development of the splenial lesion.

The transient reversibility of the diffusion deficit on MRI is what makes MERS distinct from other types of cytotoxic edema, such as that which occurs after acute infarction. Various hypotheses have been put forth to explain this property, including the fact that the corpus callosum is rich in high-density myelin fibres, which are more prone to developing intramyelinic edema. Hyponatremia, as seen in this patient, is thought to be part of the mechanism of splenial cytotoxicity by inducing interstitial edema: acute hypotonic hyponatremia results in entry of water into the brain, resulting in intramyelinic edema in the corpus callosum, which is dense in myelin [8]. Another possible explanation is that the splenial lesion consists of inflammatory infiltrates, which are either directly caused by viral antigens or indirectly via antibodies that target splenial axons [9].

In terms of differential diagnoses, it is worth noting that transient isolated corpus callosal lesions have been reported in several non-infectious conditions, including epilepsy, malnutrition, vitamin B12 deficiency, high altitude edema, alcohol poisoning, eclampsia, carbon monoxide poisoning, and migraine [5]. Acute Disseminated Encephalomyelitis (ADEM) also presents with alteration of consciousness, focal neurological signs, and seizures, following a viral infection or vaccination. The difference is that with ADEM, the MRI finding is multifocal subcortical white matter enhancement [9,10,11]. Other possible differentials for splenial lesions include demyelinating diseases such as multiple sclerosis, Marchiafava-Bignami disease, metronidazole-induced encephalopathy, CNS lymphoma, extrapontine myelinolysis, and microvascular ischaemia, which can be excluded by clinical presentation, tempo, and neuroimaging findings [12,13,14]. As this case demonstrates, the timely diagnosis of MERS can engender a favourable prognosis in a patient with acute altered mental status with a viral prodrome.

There are flaws with our approach that will require further study. As this is a single case report, which inherently limits the generalizability of the findings, we lack the statistical power to establish causal relationships or broader clinical implications. This limitation could be mitigated by comparing the case with existing literature, but our reliance on a single patient restricts the ability to draw definitive conclusions about the prevalence or typical presentation of MERS in patients with Influenza B. Furthermore, while we discuss potential management strategies that can be employed, we have not critically evaluated their efficacy. For example, it remains unknown whether the use of antiviral therapy, corticosteroids, or supportive care directly contributed to the patient’s recovery or if the improvement was simply due to the self-limiting nature of MERS. Comparing the progress of the patient reported here with other similar cases in the future may provide context for the effectiveness of the interventions used. Finally, the discussion on cytokine storms and how splenial lesions can be caused by hyponatraemia is exploratory, necessitating a deeper exploration into the underlying pathophysiological mechanisms. To this end, emerging research on biomarkers can aid in the early detection of MERS. For example, elevated levels of cytokines such as IL-6 and IL-10 have been associated with the inflammatory cascade seen in MERS, suggesting their potential utility as prognostic indicators [4,15]. Additionally, while most cases of MERS resolve spontaneously with supportive care, understanding the underlying pathophysiology can guide therapeutic decisions, such as the use of corticosteroids or intravenous immunoglobulin in severe cases [13,15]. This comprehensive approach, brought forth in this report, contributes to the broader understanding of MERS and its management in clinical practice.

## Figures and Tables

**Table 1:** Clinical Summary Table.

Category	Finding
Temperature	38.9 °C (febrile)
Heart Rate	110 beats/min (tachycardic)
Neurological Status	Confused, disoriented to time/place/person
Meningism	No meningism
Muscle Power Grading	Upper limbs 5/5, Lower limbs 4+/5 (pain-related)
Tone, Reflexes, Gait, Sensory	Unremarkable
Lymphocyte Count	Lymphopenia
C-Reactive Protein	168.6 mg/L
Sodium	125 mmol/L (hyponatremia)
Arterial Blood Gas	Mixed metabolic acidosis with partial respiratory alkalosis
Chest X-ray	Basal infiltrates
CT Brain	No masses, stroke, or hemorrhage
Serum and Urine Toxicology	Negative for illicit drugs

**Table 2:** Diagnostic criteria for MERS.

Criteria	Description
Onset	Neuropsychiatric symptoms, abnormal speech and/or behaviour, altered consciousness, seizures, within 1 week of fever onset
Recovery	Complete recovery without sequelae within 10 days of neuropsychiatric symptoms onset
Imaging (Acute Phase)	Hyperintense lesion in splenium of corpus callosum, mild T1 and T2 signal changes
Lesion Distribution	Lesion may symmetrically affect entire corpus callosum and cerebral white matter
Lesion Resolution	Lesion disappears within 1 week, no residual signal changes or atrophy

## Data Availability

All data supporting the findings of this case report are contained within the article. Further information may be available from the corresponding author upon request.

## Abbreviations

The following abbreviations are used in this manuscript:

DWI	Diffusion Weighted Imaging
ADC	Apparent Diffusion Coefficient
MERS	Mild Encephalopathy with Reversible Splenial lesion
MRI	Magnetic Resonance Imaging
